# The Effect of Omega-3 Fatty Acids on Sarcopenia: Mechanism of Action and Potential Efficacy

**DOI:** 10.3390/md21070399

**Published:** 2023-07-13

**Authors:** Atiporn Therdyothin, Nacharin Phiphopthatsanee, Masoud Isanejad

**Affiliations:** 1 Department of Musculoskeletal Ageing and Science, Institute of Life Course and Medical Sciences, University of Liverpool, Liverpool L7 8TX, UK; 2 Department of Orthopedics, Police General Hospital, Bangkok 10330, Thailand; 3 South London and Maudsley NHS Foundation Trust, London SE5 8AZ, UK

**Keywords:** omega-3 fatty acid, polyunsaturated fatty acid, fish oil, muscle mass, muscle strength, muscle function, muscle protein synthesis, sarcopenia, ageing, anti-inflammation

## Abstract

Sarcopenia, a progressive disease characterized by a decline in muscle strength, quality, and mass, affects aging population worldwide, leading to increased morbidity and mortality. Besides resistance exercise, various nutritional strategies, including omega-3 polyunsaturated fatty acid (n-3 PUFA) supplementation, have been sought to prevent this condition. This narrative review summarizes the current evidence on the effect and mechanism of n-3 PUFA on musculoskeletal health. Despite conflicting evidence, n-3 PUFA is suggested to benefit muscle mass and volume, with more evident effects with higher supplementation dose (>2 g/day). n-3 PUFA supplementation likely improves handgrip and quadriceps strength in the elderly. Improved muscle functions, measured by walking speed and time-up-to-go test, are also observed, especially with longer duration of supplementation (>6 months), although the changes are small and unlikely to be clinically meaningful. Lastly, n-3 PUFA supplementation may positively affect muscle protein synthesis response to anabolic stimuli, alleviating age-related anabolic resistance. Proposed mechanisms by which n-3 PUFA supplementation improves muscle health include 1. anti-inflammatory properties, 2. augmented expression of mechanistic target of rapamycin complex 1 (mTORC1) pathway, 3. decreased intracellular protein breakdown, 4. improved mitochondrial biogenesis and function, 5. enhanced amino acid transport, and 6. modulation of neuromuscular junction activity. In conclusion, n-3 PUFAs likely improve musculoskeletal health related to sarcopenia, with suggestive effect on muscle mass, strength, physical performance, and muscle protein synthesis. However, the interpretation of the findings is limited by the small number of participants, heterogeneity of supplementation regimens, and different measuring protocols.

## 1. Introduction

Sarcopenia is an age-related decline in muscle strength, quality, and mass [1]. It is estimated to affect more than 50 million people globally [2]. Not only does it affect musculoskeletal function, leading to falls and fractures [3,4], but it also progresses toward the deterioration in physical function [1], cardiovascular diseases [5], chronic respiratory diseases [6], cognitive impairment [7], dependence [8] and ultimately, increased mortality [9]. The condition undoubtedly increases hospitalization and healthcare costs tremendously with an estimated annual excess cost of £2.5 billion in the United Kingdom [10]. Likewise, the community-dwelling elderly with sarcopenia have more than 2-fold higher direct healthcare costs compared to those without sarcopenia [11]. According to the European Working Group on Sarcopenia in Older People, sarcopenia is diagnosed by the presence of low muscle strength (handgrip strength < 27 kg in men, and <16 kg in women, or chair stand > 15 secs for 5 rises), and reduced muscle quantity (appendicular skeletal muscle mass < 20 kg in men and <15 kg in women, or appendicular skeletal muscle mass/height^2^ < 7.0 kg/m^2^ in men or <5.5 kg/m^2^ in women. The resulting reduction in physical performance indicates its severity, with severe sarcopenia being defined by a reduction in gait speed to 0.8 m/s, short physical performance battery (SPPB) ≤ 8, time-up-and-go test (TUG) > 20 s, and 400-m walk test of ≥6 min [1].

In healthy adults, muscle mass preservation relies on a dynamic balance of muscle protein synthesis (MPS) and muscle protein breakdown (MPB), which oscillates in a dynamic equilibrium of a “fasted-loss/fed-gain” cycle [12]. Resistance exercise and adequateintake of high-quality protein containing essential amino acids, especially leucine, transiently optimize MPS, while fasting can escalate MPB [13]. MPB is also increased in aging due to physiologic and hormonal changes, as well as chronic low-grade inflammation and decreased physical activity that accompany aging [14]. MPB is also increased in various chronic muscle wasting diseases, such as cancer, cachexia, chronic kidney disease, heart failure, and chronic respiratory disease, etc. [15,16,17]. Moreover, aged muscles are less sensitive to exercise and nutrient availability which are strong anabolic stimuli. The resulting anabolic resistance makes the preservation of muscle mass a challenge for the elderly [14,18]. Without disease-specific interventions, resistance exercise remains the primary remedy against sarcopenia [19,20]. Nevertheless, considering the elderly’s limited capacity to engage in physical activities, achieving the required amount of training can be problematic [21]. Hence, developing treatment strategies to increase or maintain the quantity and strength of muscles and overcome anabolic resistance may aid in the perseverance of independence and quality of life. Omega-3 polyunsaturated fatty acid (n-3 PUFA) supplementation has gained increasing interest due to its anti-inflammatory properties [22] and biologically plausible mechanism to promote MPS by augmenting the anabolic response to hyperinsulinemia-hyperaminoacidemia and increasing protein kinases in related signaling pathways [22,23,24]. This is supported by promising results from animals [25,26] as well as clinical studies [27,28].

Comprehensively gathering the data from recent meta-analyses with updated findings from novel RCTs, this narrative review summarizes key findings on the effect of n-3 PUFA supplementation on each component of sarcopenia diagnostic criteria. The effect of n-3 PUFA on MPS will also be discussed. Plausible mechanisms by which n-3 PUFA benefits musculoskeletal health are also delineated. This narrative review will provide up-to-date and clinically relevant evidence which will aid clinical decision-making and highlight the gap in current knowledge in this field.

## 2. n-3 PUFA

n-3 fatty acids comprise a group of various fatty acids containing a double bond between the third and the fourth carbon atoms from the methyl end [29]. n-3 fatty acids can be categorized into monounsaturated fatty acid (MUFA) and polyunsaturated fatty acid (PUFA), depending on the number of double bonds in their chemical structures [30]. These fatty acids can be also classified by the number of carbon atoms, with short chain containing 6 or fewer carbon atoms, medium chain 12 carbon atoms, long chain fatty acid 14–18 carbon atoms, and very long chain containing 20 or more carbon atoms. Long-chain n-3 PUFA is the majority of n-3 PUFA available in food [31].

While the human body can synthesize n-3 PUFA from alpha-linolenic acid using desaturase and elongase enzymes, this endogenous production of n-3 PUFA only accounts for about 10% of daily PUFA requirement, which is not adequate for the body [32]. Considered an essential fatty acid, n-3 PUFA are mainly derived from external food sources and supplementation [33]. Two types of commonly occurring long-chain n-3 PUFAs in food are eicosapentaenoic acid (EPA) and docosahexaenoic acid (DHA) [34], both of which can be found in varying quantities and proportions in marine products. Microalgae oil, a rich and primary source of both EPA and DHA production, is a suitable source of n-3 PUFA for vegetarians [35,36]. Some marine invertebrates, such as oysters, squids and octopus can also synthesize these fatty acids [37,38]. Other marine fishes, such as herring, wild sardine, and mackerel, that feed on these n-3 PUFA producing algae and molluscs are also good sources of dietary n-3 PUFA [31,39]. n-3 PUFA can also be found in the roes of these oily fishes, such as salmon roe [39]. In spite of its availability in various sources, the dietary intake of n-3 PUFA was estimated to be 0.17 g per day, which only equates to about 10–15% of the recommended daily intake in adults over 50 years old [40]. Hence, products containing fish oil, cod liver oil, tuna oil, krill oil, and algal oil, are often used as n-3 PUFA supplementations [31].

n-3 PUFAS have been shown to benefit human health in various ways including metabolic issues [30], cardiovascular health [34], and muscle health [41]. Many of these benefits are largely attributable to n-3 PUFA’s anti-inflammatory properties [42]. Metabolic issues are widely known to be associated with inflammation [30]. n-3 PUFA has been suggested to ameliorate insulin resistance through improved mitochondrial function and modulation of phospholipid membranes [43]. It is also positively correlated with serum adiponectin level, exhibiting protective effects in obese individuals in decelerating the progression of cardiovascular disease and metabolic syndromes [44]. With its cardioprotective effects through blood lipids modulation, vasodilatory effect, blood pressure and heart rate modulation, lowered platelet aggregation, and a reduction of pro-inflammatory biomarkers, n-3 PUFA has been suggested as an intervention for primary and secondary preventions of cardiovascular disease [34,44]. There has been growing interest in the beneficial effects of n-3 PUFA in the prevention and treatment of sarcopenia in the elderly [45], which will be further discussed in detail.

## 3. Effect of n-3 PUFA Supplementation on Muscle Mass and Volume

Several meta-analyses suggested the positive effects of n-3 PUFA supplementation on the preservation of muscle mass. Although the results remain largely inconclusive and recommendations on the dosage are currently far from clear, dose-dependent effects have been suggested. 

A recent meta-analysis found a small increase in skeletal muscle mass of 0.33 kg after n-3 PUFA intervention compared with control in the elderly aged 60 years or above [46]. The estimation was pooled from five small studies (n = 103 for n-3 PUFA groups, and n = 99 for control groups) with low heterogeneity. Specifically, the improvement was only found in participants receiving over 2 g per day of n-3 PUFA with a pooled effect of 0.67 kg, suggesting the dose-effect relationship. However, the findings should be interpreted with caution as one of the included studies used alpha-linolenic acid supplementation as opposed to n-3 PUFA [47]. The meta-analysis also included n-3 PUFA targeted diet intervention with an instructed ratio of n-6 to n-3 PUFA ratio [48,49]. Hence, the real effect of n-3 PUFA as supplementation was obscured. Furthermore, the subgroup analysis in studies with over 2 g per day of n-3 PUFA only involved two studies with limited sample sizes [50,51], one of which was in patients with lung cancer receiving chemotherapy with profoundly different physiology from the general geriatric population. Still, the positive effects of n-3 PUFA were corroborated in another meta-analysis which compiled eight RCTs with 406 older participants [52]. The subjects, nonetheless, mainly had significant comorbidities, for example, cancer [53,54], chronic obstructive pulmonary disease [55,56], and morbid obesity undergoing surgery [57]. These patients were already subjected to chronic inflammation [58], which probably accounted for the exaggerated effect of n-3 PUFA. Moreover, the studies differed significantly in the form of n-3 PUFA supplementation, ranging from fish oils to oral multi-nutritional supplements. The duration varied vastly from 12 days to four months. One study was performed exclusively during hospitalization [55], and another did not specify the dose of n-3 PUFA [54]. However, a sensitivity analysis was not performed to exclude the effects of these methodology discrepancies.

In contrast, Cornish et al. performed a meta-analysis analyzing the effect of n-3 PUFA supplementation on lean body mass reported in 10 RCTs from 433 healthy older adults [59]. There was no difference between n-3 PUFA supplementation compared to control regardless of resistance exercise training. The meta-analysis, however, used lean body mass as opposed to skeletal muscle mass, which is considered a more sensitive and accurate indicator of body functional muscle mass [52]. Due to the variability in the study protocols, a subgroup analysis should also be performed to further explore the effects of different doses and durations of supplementation.

Lately, Dalle et al. found that neither resistance exercise training alone nor in combination with n-3 PUFA (410 mg DHA + 540 mg EPA) resulted in a gain in leg muscle volume and muscle anabolic sensitivity markers in the elderly with osteoarthritis [60]. However, interleukin-6 (IL-6), an important proinflammatory marker, was decreased, in parallel with a decreasing trend in p65NF-κB, a pro-inflammatory transcription factor, in participants receiving n-3 PUFA. The study, nevertheless, had a small sample size consisting of only 23 individuals. More recently, in a larger RCT involving 63 healthy older adults, six months of 3.9 g daily n-3 PUFA supplementation (300 mg DHA + 675 mg EPA) did not outperform placebo in increasing the leg lean mass and whole-body amino acid metabolic response to exercise [61]. Furthermore, mitochondrial respiration, adenosine triphosphate (ATP) production, and reactive oxygen species (ROS) production remained unchanged. It is worth noting that both studies had relatively short study periods, considering anabolic resistance impeding muscle gain in the elderly. In addition, it is unclear whether the exercise intervention in these studies were sufficient per se to stimulate muscle synthesis in the elderly.

Overall, n-3 PUFA supplementation may have small effects in improving muscle mass via anti-inflammation and increasing protein synthesis which will be further discussed [23,62]. Although dose-dependent relationship was suggested, the effects of different durations of supplementation were inconclusive due to high heterogeneity in research protocol, dose, duration, and form of supplementation as well as the lack of subgroup analysis. Nevertheless, since considerable duration is required to yield a detectable increment in muscle mass, longer duration of treatment should presumably be associated with greater effect. Greater effects were suggested in the population group with chronic wasting conditions and prolonged inflammation, such as cancer and chronic respiratory diseases. This requires further investigations to confirm the effect size in each group of participants, and to identify factors associated with greater benefit.

## 4. Effect of n-3 PUFA Supplementation on Muscle Strength

To date, meta-analyses on the n-3 PUFA and muscle strength have yielded conflicting results due to protocol heterogeneities such as pooling muscle strength measurement results from different muscles (as opposed to analyzing data from each parameter of muscle strength individually) or pooling outcomes of different forms of omega-3 supplementation. Moreover, these meta-analyses did not measure muscle strength as a primary outcome. Findings from randomized controlled trials are also limited by sample size and the analysis of outcomes from different supplements.

A recently published meta-analysis compared the effect of n-3 PUFA supplementation on lower and upper body strength in healthy elderly [59]. Lower body strength ameliorated after n-3 PUFA supplementation, and so did upper body strength after removing studies with a high risk of bias. While the number of pooled participants was large, this finding should be interpreted with caution as the indicators of muscle strength varied across studies, i.e., isometric torque, isokinetic torque, leg press, and one repetition maximum (1-RM), handgrip strength and chest press, reflecting different aspects of muscle strength and recruitment of different muscle fascicles [63,64,65]. Moreover, each test of muscle strength also differs in its complexity, which results in a varying degree to which training and familiarity to the test can affect the strength improvement [60]. Therefore, these parameters might be too dissimilar to be pooled into one analysis based on rough anatomical regions.

Separately analyzing each parameter of muscle strength, Bird et al. initially reported no significant effect of n-3 PUFA supplementation on handgrip strength from 14 studies [52], but later favored n-3 PUFA after removing two outliers which shows nonsignificant results [66,67]. The studies varied greatly in their duration of intervention, ranging from 26 days [68] to 3 years [69]. A subgroup analysis based on duration would have provided a better understanding. Three studies used mixed nutritional supplements containing n-3 PUFA and thus should be removed in sensitivity analysis [67,70,71]. Another meta-analysis failed to reveal the benefit of n-3 PUFA on handgrip strength in healthy older adults [46]. However, it was limited by the low number of included studies (k = 3) and outcome heterogeneity. One of the studies used mixed oral supplements as opposed to n-3 PUFA alone [72] and hence should be excluded in the sensitivity analysis. Similarly, handgrip strength was found to increase significantly with increasing dietary n-3 PUFA in another meta-analysis [73]. However, the result was pooled from only two studies, one of which combined calcium, gamma-linoleic acid, and EPA as the supplement, as opposed to conventional EPA and DHA [74]. Moreover, muscle strength was not the primary outcome in this meta-analysis.

The effect of n-3 PUFA supplementation on 1-RM maximum chest press pooled from three studies showed a non-significant negative trend [46]. Again, the analysis included a study using mixed oral supplements [72], which should otherwise be excluded from the sensitivity analysis.

Quadriceps maximal voluntary capacity was estimated from 329 participants in 10 studies, mainly involving healthy elderly samples [46]. The analysis revealed a significant increase in quadriceps maximal voluntary capacity in favor of n-3 PUFA. On the contrary, there was no improvement in 1-RM leg strength. Again, the number of included studies was rather small (k = 2), with one of them using alpha-linoleic acid instead of n-3 PUFA [47], and another prescribing healthy diet plan, instead of direct n-3 PUFA supplementation [49]. The analysis, therefore, did not represent the real effect of n-3 PUFA.

More recent trials showed conflicting results. A study in 32 chronic obstructive pulmonary disease patients randomized to receive n-3 PUFA supplementation (1400 mg DHA + 2100 mg EPA, or 1000 mg DHA + 1500 mg EPA) or placebo found no change in handgrip strength after four weeks [75]. However, the trial was limited by a short follow-up period and a small sample size. The participants had preserved muscle mass, which perhaps veiled the effect of n-3 PUFA. Eight weeks of vibration and resistance exercise training combined with whey protein and n-3 PUFA supplementation (1397 mg DHA + 749 mg EPA) significantly raised muscle power during chair rise in healthy older men but not women [76], presumably due to different gender-based responses to training and fewer motor units in females [77]. The improvement in muscle strength coincided with increased serum insulin-like growth factor 1 (IGF-1), which stimulates MPS via mechanistic target of rapamycin complex 1 (mTORC-1) signaling pathway [54,78], and also a significant reduction in pro-inflammatory cytokines, implying an anti-inflammatory effect of n-3 PUFA on skeletal muscle health. Longer-term trials, however, revealed no increase in muscle strength in 107 frail elderly after 24 weeks of supplementation of leucine-enriched protein with n-3 PUFA (1100 mg DHA + 800 mg EPA) [79].

In conclusion, n-3 PUFA supplementation seems to improve muscle strength as evident in the meta-analyses, although findings should be treated with caution due to protocol heterogeneities in the studies included. Moreover, each parameter of muscle strength may respond differently due to differences in the nature of muscle fascicles. While lower body muscle strength may also require a combination of vibration, RET, and protein supplementation, evidence supports the benefit of supplementation on hand grip strength, which is the main parameter used in both initial screening and diagnosis of sarcopenia. Moreover, handgrip strength is a strong predictor of patient outcomes, including hospital length of stay, limitation in activities of daily living, quality of life, and also all-cause mortality in the elderly. It is also related to the strengths of other body parts. Still, it remains unclear which intervention is most beneficial for each individual muscle. A comparison between n-3 and n-3 plus other nutrients will also help to elucidate this. More robust meta-analyses investigating the effect of dose and duration of n-3 PUFA supplementation are required.

## 5. Effect of n-3 PUFA Supplementation on Physical Performance

Two recently published meta-analyses of RCTs in older individuals revealed that n-3 PUFA supplementation significantly improved TUG by 0.29 s and 0.30 s respectively [46,59], although the latter was limited by the small number of pooled participants of only 73 receiving n-3 PUFA and 63 controls. Moreover, both changes, albeit statistically significant, were arguably too little to be clinically significant.

Walking speed was unaffected by n-3 PUFA supplementation in a meta-analysis of 10 studies in healthy older individuals, regardless of additional exercise intervention, although there was no subgroup analysis based on other parameters such as supplementation regimen [59]. The findings might be undermined as two of the included studies did not directly compare pure n-3 PUFA with passive placebo; one used n-3 PUFA plus vitamin E in the intervention arm [60], and the other used active placebo containing various fatty acids [80]. Another meta-analysis in older adults revealed similar results. Since the included studies had high heterogeneity, a subgroup analysis based on two studies was performed and found an increase in walking speed when supplementation period was over six months. However, one of the two studies compared n-3 PUFA enriched multi-nutritional supplement and placebo, instead of pure n-3 PUFA, which might impact the effect of n-3 PUFA in an unpredictable way [54].

Only one meta-analysis assessed 30-s sit-to-stand performance pooled from six studies with 230 participants [59]. The main analysis showed 1.93 s improvement with n-3 PUFA supplementation. The studies had high heterogeneity, with only one RCT with a high risk of bias showing a large improvement [81], and the effect was no longer significant when the study was removed.

Eight weeks of vibration and resistance exercise training with whey protein and n-3 PUFA supplementation (1397 mg DHA + 749 mg EPA) significantly improved gait speed but not chair rise time [76]. Gait speed difference, however, was very small (0.01 m/s, *p* = 0.024), and hardly yielded any clinical importance. A longer period of supplementation might yield more applicable results. The long-term effect of n-3 PUFA supplementation in the community-dwelling elderly with low gait speed or limited instrumental activity of daily living was assessed in a large-multicenter RCT which compared multiple physical performance parameters across four parallel groups: n-3 PUFA supplementation (800 mg DHA + 225 mg EPA), n-3 PUFA supplementation with lifestyle intervention, placebo alone, and lifestyle intervention alone (n = 1680) [69]. Six months of low-dose n-3 PUFA supplementation failed to improve repeated chair stand test, walking speed, SPPB, and balance. It might be noteworthy that the study was originally designed to investigate the effect of n-3 PUFA in the prevention of cognitive decline. Similarly, six months of n-3 PUFA (1100 mg DHA + 800 mg EPA) plus leucine-enriched protein did not result in any improvement in SPPB, walking speed, TUG, and chair stand test in the elderly with reduced muscle mass or strength compared to placebo, although the effect of n-3 PUFA alone was not evaluated [79].

In conclusion, n-3 PUFA fatty acid supplementation improved walking speed and TUG test in the meta-analyses. Both are important parameters in the assessment of sarcopenia severity [1]. Gait speed is a quick and easy test to perform in clinics and is also a predictor of poor quality of life and increased mortality in both community-dwelling and diseased elderly [82]. Likewise, poor TUG results are associated with frailty and higher mortality in healthy older adults [83] and those with chronic respiratory diseases [84]. However, the changes caused by n-3 PUFA supplementation were arguably too small to yield clinical significance. Some findings suggested that a longer duration of supplementation might yield more significant results. Pooled results of SPPB, which covers various domains of physical performance, might be more sensitive and helpful in understanding the effect of n-3 PUFA supplementation on physical performance.

## 6. Effect of n-3 PUFA on MPS

Research on whether n-3 PUFA supplementation enhances MPS and anabolic response yielded controversial results, and meta-analysis on the topic is still lacking. Interpretations of available findings are challenging due to various methodological limitations.

**Figure 1:**
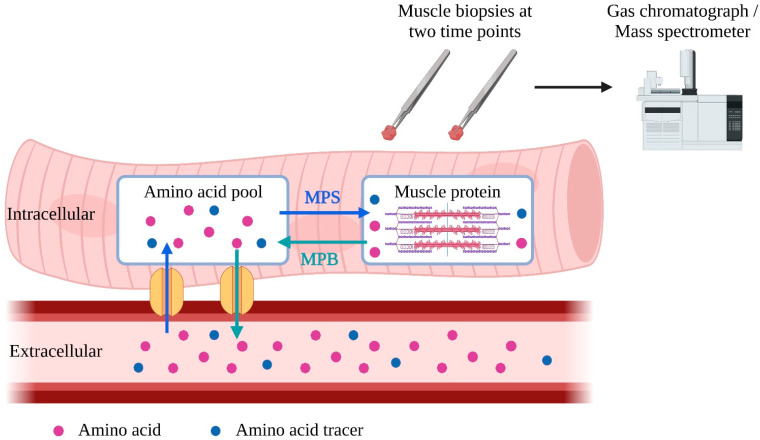
Schematic representation of measurement of fractional synthetic rate (FSR). To measure muscle protein synthesis (MPS), tracer amino acids can be introduced into the bloodstream through injection or labeling from ingested deuterated water. These tracer amino acids are taken up by the intracellular amino acid pool and used for MPS. To calculate FSR, the amount of tracer amino acid incorporated into muscle protein per unit of time is analyzed in two muscle samples. MPS = muscle protein synthesis; MPB = muscle protein breakdown. The blue arrows represent the incorporation of amino acids into the intracellular muscle pool and intracellular protein. The green arrows represent the release of amino acids of muscle protein and intracellular amino acid pool into the blood stream.

While in vivo MPB measurement remains more complicated and invasive, MPS is commonly measured in research as fractional synthetic rate (FSR) [85]. After being injected into the bloodstream, isotope-labeled amino acid tracers enter muscle cells, where they are incorporated into muscle proteins during MPS [86,87]. Two subsequent muscle biopsies are taken to determine the rate of incorporation of amino acid tracers into muscle protein to calculate FSR [88] (Figure 1). Alternatively, it is possible to use deuterium-labeled alanine produced from ingested deuterium-labeled as tracers, enabling a more extended period for FSR measurement [89].

Among the earliest clinical trials on MPS and n-3 PUFA were studies by Smith et al. which demonstrated that although n-3 PUFA supplementation (1500 mg DHA + 1860 mg EPA) did not alter the baseline muscle protein FSR compared to placebo, it augmented MPS response to hyperaminoacidemia-hyperinsulinemia anabolic stimulation as well as increased the expression of mTORC-1 signaling pathway. This effect was more evident in healthy young men compared to older individuals (50% vs. 30%) [23,28]. The increase in MPS was consistent with the increase in mTORC-1 signaling pathway expression. Positive effects of n-3 PUFA supplementation (2030 mg DHA + 2970 mg EPA) were also demonstrated in other population groups. n-3 PUFA supplementation increased the myofibrillar protein FSR in healthy young women before, during, and after leg immobilization compared to placebo, resulting in a better recovery of muscle disuse atrophy after two weeks of immobilization [90]. High dose n-3 PUFA (1400 mg DHA + 2100 mg EPA) in patients with chronic obstructive pulmonary disease improved anabolic response with approximately 15% increase in net protein synthesis [75]. Short-term effects of both high and low doses n-3 PUFA supplementation (1000 mg DHA + 1500 mg EPA) in reducing protein breakdown were also demonstrated. Finally, after 4 months of supplementation with n-3 PUFA (1200 mg DHA + 2700 mg EPA), mitochondrial and sarcoplasmic FSR were increased before exercise, while mitochondrial and myofibrillar FSR increased post-exercise [91]. The increase in MPS was parallel to the upregulation of genes that promote protein synthesis and protein folding and assembly, as well as the downregulation of myostatin, which is responsible for MPB, and other atrophy-related genes. The change in protein metabolism and genetic expression after exercise might be attributable to the reduction of anabolic resistance mediated by transcriptional changes and the alteration of anabolic signaling proteins [91,92].

On the contrary, other studies did not reiterate these results. A randomized controlled trial showed no significant increase in the FSR in healthy young men who had n-3 PUFA supplementation (900 mg DHA + 3500 mg EPA) compared to the control group having coconut oil, although the results should be interpreted with caution as the baseline FSR was not measured or recorded [93]. The lack of significant positive effects was also demonstrated in the independently living elderly after 6 months of n-3 PUFA supplementation (1200 mg DHA + 2700 mg EPA) [61]. In another study, 18 weeks of n-3 PUFA supplementation (600 mg DHA + 2100 mg EPA) contributed to a significant increase in muscle strength and quality in elderly females in the absence of an increase in MPS [27]. Another RCT failed to manifest the benefit of n-3 PUFA (1100 mg DHA + 800 mg EPA) and leucine-enriched protein supplementation, compared to leucine-enriched protein alone or placebo, in increasing MPS [79]. However, MPS was measured in only 40% of the participants. In a cohort who had hemodialysis with chronic inflammation, 2.9 g of n-3 PUFA supplementation (2:1 ratio of EPA:DHA), when compared to placebo, mitigated forearm muscle protein breakdown without significant effect on whole body protein metabolism [94]. Since the study was conducted in hemodialysis patients with many physiologic disturbances, the result might not truly represent the general geriatric population.

Overall, n-3 PUFA supplementation may positively affect MPS response to anabolic stimuli, offering a potential remedy for anabolic resistance. However, existing trials were mainly limited by the small numbers of participants and the heterogeneity of n-3 PUFA regimen, including the types of amino acid tracers, the duration between two muscle biopsies, and the physiologic condition under which muscle biopsies were taken. The lack of complete evaluation of MPS, including the lack of baseline measurement in one study also poses challenges in data interpretation. More rigorous trials with consistent outcome measures are needed to elucidate the relationship between n-3 PUFA and MPS.

## 7. Mechanism of n-3 PUFA on Skeletal Muscle Health

**Figure 2:**
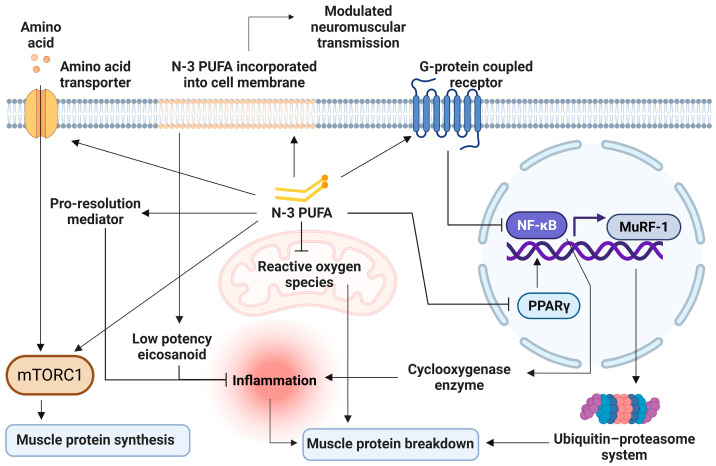
Cellular mechanisms of n-3 PUFA on skeletal muscles. n-3 PUFA displaces n-6 PUFA on membranes, resulting in less inflammatory mediators production, and produces pro-resolution mediators. It also reduces cyclooxygenase production through the inhibition of nuclear factor kappa B (NF-κB). Decreased inflammation leads to decreased muscle protein breakdown. NF-κB inhibition also suppresses the ubiquitin-proteasome system through the downregulation of the muscle ring finger-1 (MuRF-1) gene. n-3 PUFA activates the mTORC-1 signaling pathway, stimulating muscle protein synthesis by phosphorylation of involving protein kinases. n-3 PUFA increases amino acid transporter expressions, allowing more substrates for protein synthesis. n-3 PUFA improves mitochondrial function and reduces free radicle production, which leads to muscle breakdown. mTORC-1 = Mechanistic target of rapamycin complex 1; MuRF-1 = muscle ring finger-1; NF-κB = Nuclear factor kappa B; n-3 PUFA = omega-3 polyunsaturated fatty acid; PPAR-γ = peroxisome proliferator-activated receptor gamma.

To date, meta-analysis on the mechanism of n-3 PUFA on skeletal muscle health remains scarce. RCTs in animals and human have suggested different fragments of entwining mechanisms on how n-3 PUFA can benefit skeletal muscle health and function, which are 1. Reducing inflammation, 2. Increasing MPS through mTORc1 pathway, 3. Decreasing MPB through ubiquitin-proteasome system (UPS) and autophagy lysosome system (ALS), 4. Improving mitochondrial function, 5. Increasing cellular amino acid transport, and 6. Optimizing membrane fluidity (Figure 2).

The most important mechanism is probably anti-inflammation [95]. Recently, an umbrella meta-analysis found reduced serum C-reactive protein (CRP), tumor necrosis factor α (TNFα), and IL-6 concentrations in healthy and diseased elderly participants receiving n-3 PUFA supplementation [96,97,98,99], with higher heterogeneity among middle-aged adults [27,66,80]. The anti-inflammatory effect of n-3 PUFA arose from EPA and DHA’s ability to displace arachidonic acid, a principal substrate for potent inflammatory mediators [100]. Twelve weeks of n-3 PUFA supplementation increases EPA and DHA components by approximately three folds in muscle cell membranes, while decreasing n-6 to n-3 ratios by 3 folds [101]. A higher n-6 to n-3 ratio in plasma was found to be associated with increased inflammatory markers such as CRP, IL-6, and TNFα [102]. EPA and DHA are metabolized into eicosanoids with lower inflammatory potency [22]. In addition, n-3 PUFA lessened cyclooxygenase-2 (COX-2) production via the inhibition of nuclear factor kappa B (NF-κB) [103] through the activation of peroxisome proliferator-activated receptor gamma (PPAR-γ) [104,105] and the stimulation of G-protein coupled receptor GPR120 [22,106]. The consumption of COX inhibitor was previously found to benefit skeletal muscle function [107], lower the risk of sarcopenia [108], and increase muscle fiber size in older adults [109]. However, due to its potential side effects, COX inhibitor was not prescribed for long-term use in the elderly [110]. The inhibition of NF-κB also reduces downstream stimulation of muscle ring finger-1 (MuRF-1) gene, which encodes for muscle-specific E3 ubiquitin ligase, the key enzyme of UPS, the main intracellular protein degradation machinery [65,111]. Furthermore, EPA and DHA are also substrates for pro-resolution mediators such as resolvins, protectins and maresins [112], which limit leukocyte trafficking, increase the clearance of inflammatory debris, and reduce inflammatory cytokine production [113]. While both EPA and DHA share anti-inflammatory effects, DHA has been found to suppress a wider array of inflammatory genes and cytokine responses compared to EPA in monocytes under chronic inflammation stimulation. However, their effects are similar in unstimulated conditions [114,115].

Another mechanism by which n-3 PUFA enhances skeletal muscle health is through protein kinase activation in the mTORC-1 pathway, the key pathway in muscle synthesis [116,117,118]. n-3 PUFA supplementation elevated focal adhesion kinase (FAK) expression [119], which in turn, activated the mTORC1 signaling pathway, initiating MPS [120]. Likewise, there was a 30–50% increase in MPS in response to anabolic stimuli along with a parallel increase of approximately 50% of mTORSer2448 concentrations and its downstream product in muscle cells after eight weeks of n-3 PUFA supplementation [23,24]. Microarray analyses from best responders to six months of n-3 PUFA supplementation showed decreased inhibition of the mTORC-1 signaling pathway compared to participants receiving a placebo [92].

n-3 PUFA decreases protein breakdown by UPS and ALS [121]. UPS is the major pathway in cellular protein breakdown, including myofibrillar proteins. The rate-limiting step of UPS in the muscles is the ubiquitination of two E3 ubiquitin ligases, muscle atrophy F-box (MAFbx) and MuRF1 [122]. EPA can reduce MuRF1 expression by preventing NF-κB nuclear binding, and finally suppressing muscle protein degradation [123,124]. DHA restricted UPS activity through the obstruction of proteasome catalytic sites by excessive loads of DHA-mediated oxidized proteins [125]. Six months of n-3 PUFA supplementation in older adults suppressed the expression of ubiquitin-mediated proteolysis from microarray analysis [92]. ALS is responsible for the degradation of protein complexes and malfunctioned organelles [121]. DHA was found to decrease the rate of autophagosome formation, and therefore, slow down ALS [126]. However, ALS operates at a basal rate during normal physiology, and its role in sarcopenia pathogenesis remains uncertain.

Many authors believe that when compared with DHA, EPA is responsible for improved muscle protein turnover [45,76]. Incubation with 50 micromolar EPA, but not DHA, has been shown to increase C2C12 myotubes protein synthesis and decrease protein breakdown in mice [127]. However, at higher doses (300–700 micromolar), DHA can more effectively decrease muscle protein breakdown [128]. It is noteworthy that in both studies, the dose is much higher than the doses typically used in clinical trials [45].

n-3 PUFA improves mitochondrial biogenesis and function by increasing glycolytic capacity, basal oxidative metabolism, oxygen consumption, total mitochondrial metabolism, and mitochondrial content in muscle cells [129,130,131]. An in vivo study showed that n-3 PUFA supplementation increases mitochondrial and peroxisomal density, in addition to peroxisome enzymes involved in fatty acid oxidation [131]. Older adults receiving n-3 PUFA showed significantly higher expressions of UCP3 and UQCRC1 which are the key components of the electron transport chain [92]. n-3 PUFA supplementation increases mitochondrial adenosine diphosphate sensitivity [132], leading to declined ROS production by 20–25% [133,134]. Reduced ROS emission with n-3 supplementation is in accordance with a higher rate of mitochondrial and sarcoplasmic protein synthesis [91]. It also decreases susceptibility to oxidative damage [132], which precipitates MPB [135]. Furthermore, n-3 PUFA supplementation maintains adenosine diphosphate-stimulated mitochondrial respiration and mitochondrial protein synthesis during leg immobilization, which would otherwise decrease during prolonged immobilization [90]. The finding implies the role of n-3 PUFA in preventing muscle disuse atrophy.

n-3 PUFA might improve cellular amino acid transport. Expression of L-amino acid transporter (LAT) and sodium-coupled neural amino acid transporter-2 was increased in pigs fed with diets rich in n-3 PUFA, enabling more substrate for MPS [136]. There was also a trend toward an increase in LAT-1 expression in young women receiving high dose n-3 PUFA during two weeks of leg immobilization with a slower decline of muscle mass compared to placebo (*p* = 0.06) [90].

**Figure 3:**
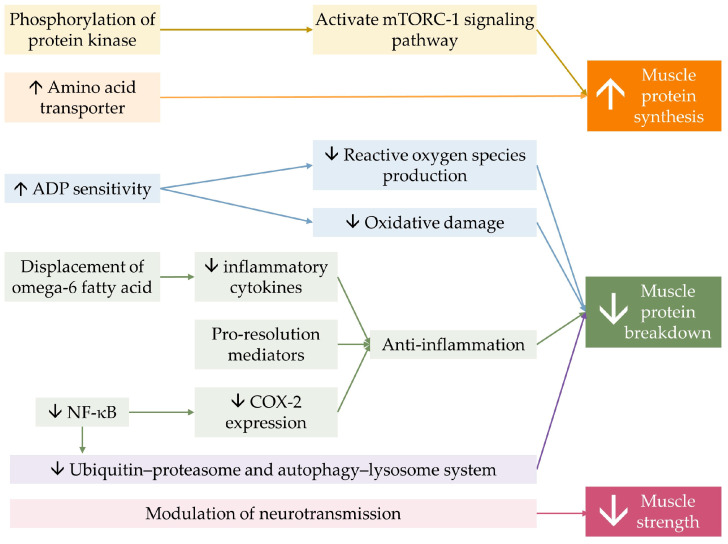
Conceptual summary of the mechanism of n-3 PUFA on musculoskeletal health. n-3 PUFA activates mTORC-1 signaling pathway, by phosphorylation of involving protein kinases. It also increases the expression amino of acid transporter, allowing more substrates for protein synthesis. n-3 PUFA improves mitochondrial function by increasing mitochondrial Adenosine diphosphate (ADP) sensitivity. n-3 PUFA displaces omega-6 fatty acid on membranes, resulting in less production of inflammatory mediators, and produces pro-resolution mediators, constituting less muscle protein breakdown. Omega-3 reduces cyclooxygenase-2 (COX-2) expression through the inhibition of nuclear factor kappa B. n-3 PUFA directly decreases intracellular protein breakdown. Together, they result in a greater muscle quantity. Lastly, n-3 PUFA modulates neurotransmission, resulting in greater muscle strength. The ↑ symbol represents an increase, while the ↓ symbol represents a decrease.

Lastly, optimizing membrane fluidity by n-3 PUFA, owing to its ability to incorporate into phospholipid bilayers of the cell membrane, could possibly influence the membrane’s property and flexibility, resulting in the modulation of neurotransmitter transmission [101,137,138]. This mechanism is believed to be attributable to DHA, which is present in higher concentrations than EPA in neuromuscular tissue [139]. n-3 PUFA supplementation combined with strength training was found to increase skeletal muscle activation levels and decrease electromechanical response time in older women, leading to greater improvement in muscle strength and functional capacity, thereby enhancing the effect of resistance training [81]. The benefit of n-3 PUFA on muscle contractility in response to neural stimuli was suspected to explain muscle strength improvement independent of muscle mass and volume [59,60,81]. Figure 3 demonstrates a conceptual diagram summarizing the effect of n-3 PUFA on musculoskeletal health.

## 8. Gap of Knowledge and Future Research Direction

Various clinical trials have attempted to explore the effect of n-3 PUFA supplementation on different aspects of musculoskeletal health. However, controversies and a large gap of knowledge still exist due to the heterogeneity of results which probably stem from different protocols of supplementation, and additional physical and nutritional intervention given alongside n-3 PUFA. There is a paucity of the data on the comparative effect of EPA versus DHA on musculoskeletal health and function since most clinical trials used combined EPA and DHA in varying proportions as their interventions without any trials directly comparing the effect of pure EPA versus pure DHA. More importantly, the difference in an individual’s response might lead to greatly varying results, especially when trials are pooled together.

Since n-3 PUFA is relatively safe and widely accessible, n-3 PUFA supplements might be a promising and cost-effective strategy for muscle preservation in the elderly. Future research focusing on how the effect of n-3 PUFA supplementation can be maximized will provide a more tangible clinical application, i.e., the most effective form and dose of n-PUFA, an optimal duration of supplementation, appropriate additional physical training, factors affecting the response to n-3 PUFA supplementation (i.e., age, gender, baseline EPA, DHA level), and conditions that will most benefit from the supplementation (healthy vs. with chronic muscle wasting diseases). The parameters used for the measurement of the effect of n-3 PUFA on muscles and physical function should be sensitive and reflect important health outcomes. Larger trials exploring the effect of n-3 PUFA on MPS should implement more homogeneous protocols of MPS measurement (i.e., the choice of amino acid tracer, the duration of measurement, and the presence of anabolic stimulation during measurement). Future rigorous meta-analyses focusing on each parameter of sarcopenia are needed. Sensitivity analyses and subgroup analyses based on the dose, duration, and types of n-3 PUFA supplementation, as well as demographic data such as gender and health status, should be performed to more clearly delineate the effect of n-3 PUFA supplementation. Meta-regression should also be done to identify the factors associated with greater benefit.

## 9. Conclusions

Sarcopenia can lead to various negative health outcomes. The diagnosis of sarcopenia relies on the reduction in muscle mass and muscle strength, with a decrease in physical function indicating severe sarcopenia. Compiling the most up-to-date evidence from meta-analyses and RCTs, this narrative review found the benefits of n-3 PUFA in various aspects of sarcopenia. n-3 PUFA supplementation provides small benefits in improving muscle mass with a possible dose-effect relationship. In general, the effect of n-3 PUFA supplementation on muscle strength was shown to be positive. Nonetheless, each parameter of muscle strength may respond differently. It also demonstrated pooled benefits on physical function, in particular walking speed and TUG test. However, the changes were too small to bear clinical significance. Longer duration of supplementation tends to be more favorable. n-3 PUFA may positively affect MPS response to anabolic stimuli. Mechanisms by which n-3 PUFA supplementation improves muscle health are 1. Anti-inflammation, 2. Increased expression of mTORC1 pathway, 3. Decreased UPS and ALS, 4. Enhanced mitochondrial biogenesis and function, 5. Improved amino acid transport, and 6. Improved neuromuscular junction activity. However, relevant trials differ profoundly in their research protocols, supplementation regimens, and outcome measurements, resulting in highly heterogeneous results. Robust meta-analyses with thorough subgroup analysis are required.

## Data Availability

This is a review paper. Data were utilized from open-access resources.
